# ACTIVE ENGAGEMENT AFTER RETIREMENT

**DOI:** 10.1093/geroni/igad104.1061

**Published:** 2023-12-21

**Authors:** Dannii Yeung, Edwin Chung

## Abstract

Retirement has been shown to accelerate the declines in physical and mental health (Dave et al., 2008) and cognitive functioning (Celidoni et al., 2017; Xue et al., 2018) due to the identity disruption and removal of a cognitively simulating environment after leaving the workforce. This necessitates a need to systematically uncover factors that could potentially buffer the negative impacts brought by retirement. This symposium aims to identify the types of self-initiated activities that can maintain post-retirement well-being through research conducted in Hong Kong and Japan. Specifically, the first paper from Chung reveals that retirees engaging in postretirement work exhibit lower life satisfaction but higher cognitive functioning than those without work. Similarly, the second paper from Katagiri also shows that work has negative impacts on life satisfaction, but such effect is buffered by civic participation. The third paper from Ho suggests that the beneficial effects of ICT use on personal mastery is more prevalent in retirees than in non-retirees. The fourth paper from Lin discloses that participation in cognitively demanding volunteering activities is associated with better cognitive and psychological functioning. These four presentations unveil various types of post-retirement activities that are beneficial to retirees, including re-engagement in the workforce, the use of technology for leisure and financial purposes, and participation in cognitively stimulating volunteering. Given that most of the pre-retirees do not have much planning on social life arrangement, findings of this symposium provide practical implications to improve psychological adjustment to this critical life event.

